# Public Engagement with Biotechnologies Offers Lessons for the Governance of Geoengineering Research and Beyond

**DOI:** 10.1371/journal.pbio.1001707

**Published:** 2013-11-12

**Authors:** Jack Stilgoe, Matthew Watson, Kirsty Kuo

**Affiliations:** 1 University College London, London, United Kingdom; 2 University of Bristol, Bristol, United Kingdom; 3 Cambridge University, Cambridge, United Kingdom; Kings College London, United Kingdom and Nikolas Rose, Kings College London, United Kingdom

## Abstract

This Perspective looks back on recent experience of public engagement with biotechnologies and asks what can be learned for the governance of another controversial emerging area—geoengineering.

**Summary**

In this paper, we reflect on our involvement in one of the first major research projects in the emerging area of geoengineering (the deliberate intervention in the planetary climate). The project, Stratospheric Particle Injection for Climate Engineering (SPICE), proposed an outdoor experiment that attracted substantial public scrutiny despite a strong consensus that the experiment posed no direct environmental risk. A programme of stakeholder engagement took place that sought a deep understanding of the views about the proposed experiment. The lessons from this experiment build on insights from public engagement with the biosciences and biotechnology. In particular, we see the importance of questions of context and purpose for scientific research. This has important implications for the governance of geoengineering research. Efforts to detach areas of research from public scrutiny by using thresholds, whether these are drawn at a particular level of environmental effect or at the doors of a laboratory, will encounter problems of public credibility. Geoengineering is unavoidably entangled in a political discussion that scientists should seek to understand and engage with.

## Introduction

In June of 1816, in a villa on the shores of Lake Geneva, Mary Godwin and three friends were kept inside by what she called as a "wet, ungenial summer." The thunderstorms and "incessant rain" she described were not a normal spell of bad weather, however. A year earlier, the eruption of Mount Tambora in Indonesia, the largest of the modern era, had thrown over one hundred million tonnes of sulphur dioxide into the mid stratosphere [1]. The sulphur dioxide reacted with atmospheric water and oxygen to produce a sulphate layer that was dispersed around the globe, dimming the incoming sunlight and leading to what became known as "the year without a summer" [2]. The failures of harvests led to food riots throughout Europe [3].

Mary Godwin went on to marry Percy Shelley, one of the other guests of the villa. The book she began writing that summer, the introduction of which contains the author's reflections on the bad weather that forced her to stay inside, became Frankenstein [4]. The thunderstorms she experienced became part of the dramatic backdrop for her story of a scientific experiment that gave birth to a monster [5].

Such climatic disruption has inspired scientific as well as literary insight. In June 1991 the eruption of Mt. Pinatubo heralded a new understanding of the climatic impacts of large volcanic eruptions. An injection five or six times smaller than Tambora [6] still managed to exert considerable influence on global climate, cooling the Earth by an average of about 0.5°C for about 2 years [7], with impacts on storms, rainfall, and international conflict [8]. The eruption was the first of its size observed by satellites, facilitating a quantum leap in our understanding of how volcanoes influence climate. Observation and quantification of the way that large volcanic eruptions reduce global temperatures by dimming sunlight has prompted some to suggest that there might be ways to artificially engineer this effect in order to counteract global warming [9].

This proposal, known as stratospheric particle injection, fits under the banner of geoengineering, defined by the Royal Society as "the deliberate large-scale manipulation of the planetary environment to counteract anthropogenic climate change" [10]. More specifically, it is an example of a subset of geoengineering proposals known as solar radiation management (SRM). Geoengineering proposals have a long history, dating back at least to the Cold War, and longer if one considers attempts at local weather modification [11]; but there has been a recent and dramatic revival of scientific and policy interest, as awareness of the scale of climate change risks has become more pronounced [12].

In this paper, we reflect on our involvement in one of the first major SRM research projects, SPICE (Stratospheric Particle Injection for Climate Engineering). We look back on recent public engagement with biotechnologies, to compare this with our own stakeholder engagement work. Questions to do with the context and wider purposes of scientific research emerge as important, but these are conventionally neglected in governance.

### Questioning Emerging Technologies

In the 200 years since it was written, Frankenstein has become the preeminent parable of technology-out-of-control. The Frankenstein myth looms over biotechnology and the biosciences in particular because it concerns the creation and manipulation of life. But its resonance goes well beyond the biosciences. The story reflects societal unease about the tension between innovation and responsibility. Its name is invoked in popular criticisms of technologies ("Frankenstein food" or, more recently, "Frankenstein Fish") and scientific irresponsibility.

Each new technology brings unique possibilities, challenges, and dilemmas. Technologies have different technical implications and different "social constitutions" [13]. And yet there are lessons from past technologies that we can apply to those currently emerging [14]. There are ways in which the social, political, and ethical concerns that may come to govern their emergence can be anticipated [15],[16].

The progress of biotechnology brings the potential for ever more intimate and disruptive interventions into human bodies and the natural environment. As previous papers in this series have described, there have been various attempts, especially in the last decade, to improve engagement between scientists and public groups on issues involving biotechnology [17]. Engagement exercises, whether with particular non-science stakeholders or members of the general public, reveal layers of societal concern with these technologies. There is typically concern with the eventual downstream risks and the ethical implications of technologies. But these things are hard to assess in advance due to the profound uncertainty that surrounds emerging technology. Public engagement typically also reveals a set of "upstream" concerns [18].

When brought into dialogue about emerging technologies, before it is clear what the risks are likely to be, members of the public will typically express concern about the trajectory of technological pathways. A report of one large public dialogue exercise on Synthetic Biology [19] drew out five questions for scientists that characterised public concerns about this nascent technology:

- What is the purpose?
- Why do you want to do it?
- What are you going to gain from it?
- What else is it going to do?
- How do you know you are right?

These questions get to the heart of the politics of emerging technologies and the foundations of public trust in scientific research. Conventional technology assessment considers the downstream products of research and innovation with a focus on technological risk and ethics [20]. More recent anticipatory governance approaches, such as "constructive technology assessment" [16], "real-time technology assessment" [15], and "responsible innovation" [21], attempt to broaden the debate to include consideration of the processes and purposes of research, in line with the five questions above. Such approaches emphasise the importance of democratic deliberation in "opening up" the technological options and trajectories for appraisal [22]. Geoengineering in general and the SPICE project in particular have become important test cases for this new mode of governance [23].

**Figure 1:**
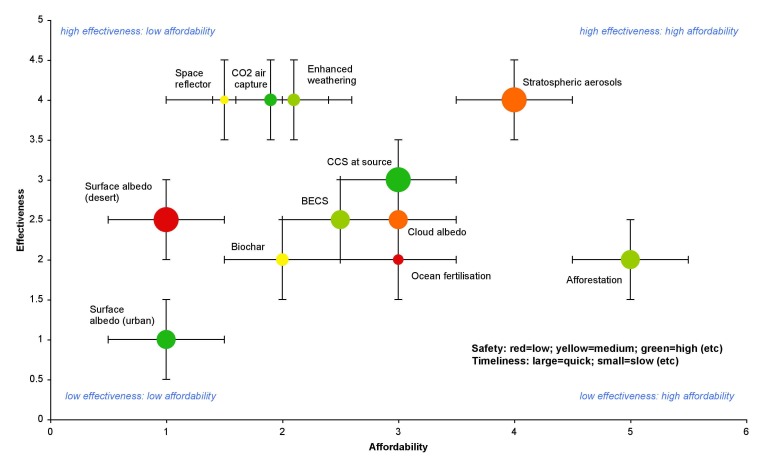
Preliminary overall evaluation of geoengineering techniques [10, p. 49].

Anticipatory governance demands a focus on the trajectories of technology, even if those trajectories are highly uncertain. As one of the first major research projects explicitly looking at geoengineering, the SPICE team are concerned about the long-term unintended consequences of our research. There is a risk that any research project that focuses on a particular technology contributes to "locking in" [24],[25] that option to the detriment of other options, including non-technological ones. The SPICE project follows an apparent, if unintended, favouritism towards stratospheric aerosols by the Royal Society in its influential report on geoengineering (Figure 1).

Stratospheric aerosol injection was determined to be the fastest, most effective, and cheapest geoengineering option, but also, when compared with Carbon Dioxide Removal, more potentially dangerous. Such assessments are crucially dependent on framing assumptions, some of which were indicated by the Royal Society [10], and characterised by profound uncertainty. Given this uncertainty, there is a danger of social science and engagement engaging in "speculative ethics," treating the technology and its implications as fully formed [26]. So while there is a clear opportunity for SPICE to provide an opportunity for public and stakeholder engagement, engagement should not be considered to be a neutral exercise [27].

### SPICE and Stakeholder Engagement

SPICE is a multidisciplinary research project aiming to investigate the possible benefits, risks, costs, and feasibility of solar radiation management by injecting reflective particles into the stratosphere. It is one of the first large SRM research projects anywhere in the world, and the first to propose an outdoor experiment. This "testbed" component was designed to investigate the dynamics of a tethered-balloon delivery system.

Recent reports from the Royal Society and others explain the scientific, social, and political complexity of geoengineering [10],[28],[29]. Scientists evaluating proposed geoengineering approaches have pointed to the possible problems that we might anticipate and others about which we are hugely uncertain [30]. Nevertheless, it is argued that the risks of not knowing whether and how geoengineering might work outweigh the risks associated with embarking on a research program [31]–[33], the latter including the possibility of distracting political attention away from climate change mitigation efforts [10]. This is in part why such emphasis has been placed on governing the technology before it is fully-formed [34].

**Figure 2:**
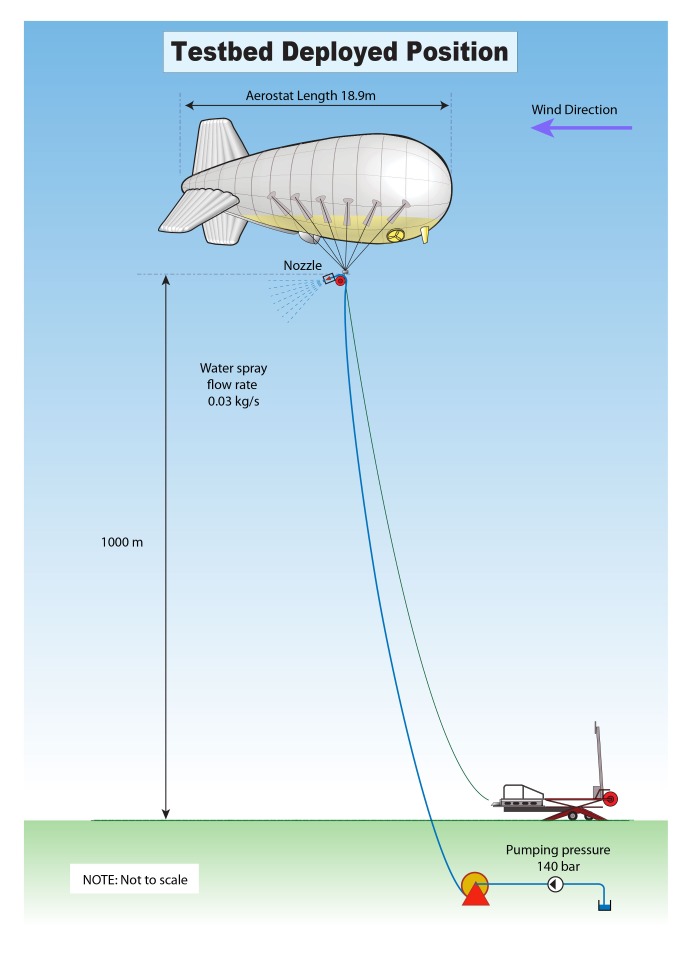
Proposed SPICE testbed.

The testbed proposed as part of the SPICE project (Figure 2) attracted substantial attention from interest groups, mainstream news, and specialist scientific media. It acted to raise the profile of geoengineering in the public sphere and the resulting debate can productively be viewed as a form of "informal technology assessment" [35]. The SPICE team, following discussion with an external "stage-gate" panel [36], sought and attempted to understand a wide range of responses to their proposed research.

In order to understand the nature of these concerns and learn lessons for the governance of geoengineering research, a programme of stakeholder engagement took place, involving 29 qualitative interviews (ranging from 30 minutes to 2 hours) with civil society organisation (environmental or humanitarian non-governmental organisation [NGO]) representatives (*n*=12), scientists (*n*=11), and other actors (*n*=6), and a workshop at which 15 participants (seven from NGOs and eight scientists and others) openly shared and discussed their concerns. The stakeholders were selected in order to provide a range of critical but constructive views on the SPICE project, with an initial sample "snowballed" [37] to form the larger group of recommended participants. This research should therefore be taken neither as quantitative nor as representative of a broader public view of SPICE or geoengineering. (Additional deliberative public engagement work has been conducted with respect to SPICE [38],[39]). The views of these stakeholders suggest that the big questions that characterise debates over genetically modified organisms and synthetic biology are pertinent too for geoengineering. In the sections that follow, quotations are taken from the interviews and workshop without attribution or coding, so as to maintain the anonymity of the participants.

### "The Imaginary Made Real": Insights From Stakeholder Engagement

Among the stakeholders around the SPICE project, there was a surprising degree of consensus about two things: first, that the SPICE testbed did not in itself pose direct environmental risks and, second, that it was nonetheless contentious and deserving of further deliberation. As we will describe in this paper's conclusion, this has important implications for governance.

Asked to explain what concerned them about the testbed, most stakeholders identified a troubling wider vision of the future:

*"It's the imaginary made real."*

*"We weren't concerned about the direct impacts of that experiment, it was the role it plays in developing the technology and bringing forward the day on which the technology will be deployed."*

*"The trial wasn't risky, but it was being done for a reason, and the reason is risky… It was clear that this wasn't pure research. The purpose was the problem."*

*"One question that is too infrequently asked is ‘why?’ It's not a specific concern about the impacts of any one experiment. It's a concern about the implications of those experiments."*

*"The purpose of the experiment is to try to figure out how to blast a material into some part of the atmosphere… the whole purpose of the experiment is to deploy geoengineering."*

The last three comments reveal a concern with the purposes of research that is hard to account for in current governance. Geoengineering, unlike previous emerging technologies such as nanotechnology and synthetic biology, is defined by its intent, its statement of purpose. So whereas with nanotechnology it has proven hard to deliberate on upstream questions of purpose [40], geoengineering presents the opportunity for a more constructive discussion.

There is an acute awareness, displayed in the first two comments above, that research could be a step onto a slippery slope. Dale Jamieson, in an early reflection on the ethics of geoengineering, warns that "researching a technology risks inappropriately developing it. Often we think of research as being quite independent of development. Unfortunately this often is not true. In many cases research leads unreflectively to development" [41, p. 333] (see also [42]). Stakeholders are therefore watchful of any interests or commitments that might lead to technological "lock-in." In the case of SPICE, the issue of intellectual property became an important consideration. A patent application relating to the balloon technology, on which two SPICE researchers, despite not holding intellectual property themselves, were listed as inventors, was made prior to the launch of the project. This coloured the views of some stakeholders:

*"It isn't just a balloon in the air any more when the patent ties it explicitly to geoengineering."*

Such concerns are predicated on an assumption that a particular geoengineering research project cannot be divorced from its political context. Research into technologies for geoengineering and the physical impacts of climate modification cannot, in the minds of many stakeholders, be conducted independently of the socio-political issues around technological deployment:

*"In geoengineering research, context is everything. You know I don't think there is a way round that. You know these are such big questions and the implications of deploying a geoengineering technique are so enormous."*

The SPICE researchers and their funders have come to terms with these implications via invited and uninvited engagement with various public groups. After initially proposing the testbed, and then having it postponed by the project's funders while stakeholder engagement work took place, the SPICE team took the decision not to proceed with the experiment, given the gaps in governance and uncertainties about intellectual property [43]. This decision prompted substantial debate and reflection in the media, within the SPICE team and among others interested in geoengineering governance [44],[45].

### Governing Geoengineering Research—Detachment and Entanglement

Until recently, it was relatively easy to categorise geoengineering research as what historian James Fleming calls "geo-scientific speculation" [11]. Following some prominent scientific and policy interventions, geoengineering has rapidly become a policy issue and a research agenda. In addition to computer modelling and laboratory studies, some SRM researchers are now proposing outdoor experiments [46]. On the Carbon Dioxide Removal side of geoengineering, a few controversial experiments in the open environment have already taken place [47],[48]. Other scientists suggest that these are premature, that research should stay indoors until we know more [49]. Discussions that have taken place under the aegis of the Solar Radiation Management Governance Initiative (an offshoot of the Royal Society report) suggest that consensus on this should not be expected soon.

Despite the absence of a direct climatic effect, the proposed SPICE testbed would have been a symbolic step in the direction of outdoor experimentation. Any outdoor experiments designed to test the efficacy or implications of geoengineering would involve some level of deployment of a technology. And, in the event of a future decision to deploy a technology to cool the earth, the huge uncertainties involved mean that the deployment would itself be experimental [50],[51]. With SPICE, stakeholders' concerns are less with the present circumstances of the testbed experiment and more with the steps that might be taken beyond it.

Research and innovation are conventionally governed by two main regimes of regulation, those of risk and ethics. The machinery for governing these things is well-established. We have risk assessments, risk registers, and processes for risk management, including the precautionary principle for areas in which risks are not well-defined. Similarly, ethics committees at universities, hospitals, and other places where research takes place are designed to safeguard the rights of participants. Among the stakeholders, SPICE was considered relatively benign on the grounds of both risk and ethics. There was a consensus that the risks of the testbed were trivial and it was approved by research ethics committees of the universities involved subject to its safety and legality. And yet the project has generated substantial controversy. We have argued in this paper that this controversy has been useful, as a pointer towards wider concerns about geoengineering.

SPICE therefore provides an indication of the limits of governance. The sorts of insights typically revealed by public engagement can be hard to build into a regulatory framework, and we lack a framework for consideration of the sort of "public ethics" [52] issues presented by geoengineering proposals. As geoengineering research gathers pace, however, it is vital to consider how governance might become more "socially robust" [53]. There is a choice between detachment and entanglement.

The growth of geoengineering research has been accompanied by calls for governance. Much of the time, "governance" is interpreted in the hard, regulatory sense of prohibition. Here, scientific freedom or the "right to research" [54] is interpreted in the sense of freedom from interference, what Isaiah Berlin calls "negative liberty." The approach suggested by Parson and Keith in a recent paper in *Science* argues for an experimental threshold [46]. Their suggestion is that, above a certain upper limit (where there is a discernable effect on the environment), there should be a ban on geoengineering experiments. They also suggest a lower limit, beneath which experiments should be allowed to take place unfettered. Others have proposed an indoor/outdoor divide [29],[49], with the premise that indoor activities are ethically justifiable, while activities outside the laboratory demand additional scrutiny. These are two arguments for detachment. We have seen through the SPICE project that such an attempt to cordon off research from public attention will be problematic, not least because there will be substantial disagreement about where the threshold should lie. Outdoor experiments that do not perturb the environment cannot be considered to be immune from potential controversy and isolated from the socio-political issues relating to deployment. Nor is indoor research insulated from the political and ethical discussion. Scientifically defined thresholds for public scrutiny, whether they are placed at particular levels of radiative forcing or at the doors of a laboratory, will not in themselves command public credibility. As Brown and Guston describe, there is another interpretation of the "right to research" that is more democratic and seeks engagement between research, politics, and the public rather than detachment [54].

One of the major lessons of public engagement with biotechnologies has been that purposes matter. Why something is being researched or developed is an issue of substantial public interest. Research is situated in, and inextricable from, its wider social context [55]. The SPICE project has been explicit about its connections with geoengineering. It has therefore attracted a good deal of attention. Other projects have engaged with geoengineering more obliquely, informing geoengineering research while ostensibly labelling themselves as climate science. By way of comparison, we might point to the E-PEACE project, which has taken place over a similar timescale to SPICE and involves an outdoor experiment in cloud formation. The E-PEACE project has clear relevance to geoengineering research but has not labelled itself as a geoengineering research project, so has avoided some of the attention attracted by SPICE.

The discussion and governance of purposes will be difficult, as Parson and Keith describe [46]. Emerging technologies can readily be reclassified as mundane if there is a fear of burdensome regulation [56]. But that should not stop us from attempting to improve the responsible governance of science. Rather than presupposing some unhelpful dichotomies, between science and society, between innovators and regulators, we could instead look to continued discussions among scientists and publics as a basis for governance, understood broadly to encompass the norms, cultures, and practices of science.

SPICE and other geoengineering projects are unavoidably entangled in the politics of geoengineering. Rather than seeking to escape these entanglements [57], scientists can instead try to understand and work with them, through processes of public and stakeholder engagement. The SPICE team, over a series of several months, have been through a programme of governance and engagement, including the "stage-gate" process imposed by the project's funders, the UK Research Councils, requiring substantial effort and occasional discomfort, but this has been ultimately worthwhile. Researchers' involvement in politicised issues involving emerging technologies demands consideration of new responsibilities, some of which have been imposed upon SPICE and some of which SPICE have invited. According to Bruno Latour, the story of Frankenstein offers a lesson here:

"Dr Frankenstein's crime was not that he invented a creature through some combination of hubris and high technology, but rather that he abandoned the creature to itself." [58]

Within geoengineering research, scientists are wrestling with the tangle of politics and ethics in which their work takes place. Scientists have been aware of the political discussion associated with geoengineering, but have understood and engaged with questions of responsibility in different ways. In 2009, a scientific conference at Asilomar began discussions on self-governance that were subsequently taken on by the Solar Radiation Management Governance Initiative [59],[60]. The Oxford principles for geoengineering governance have provided an initial basis for these discussions [61]. But there remains a gap between these abstract norms and the practice of research. The SPICE experience suggests that, while science cannot set the terms for public and stakeholder debate, history and public engagement can attune scientists to the questions that are overlooked in discussions focussing on risk. Scientists in areas of emerging science and technology are well-placed to initiate such a dialogue. But the responsibility should not fall on their shoulders alone. Responsible governance demands meaningful, early collaboration with social scientists and ethicists. Crucially, institutions, including research funders, must also take active responsibility for their own decisions.
